# Minimum initial service package (MISP) for sexual and reproductive health for women in a displacement setting: a narrative review on the Syrian refugee crisis in Lebanon

**DOI:** 10.1186/s12978-021-01108-9

**Published:** 2021-03-08

**Authors:** Dana Nabulsi, Maya Abou Saad, Hussein Ismail, Myrna A. A. Doumit, Fatima El-Jamil, Loulou Kobeissi, Fouad M. Fouad

**Affiliations:** 1 Refugee Health Program, Global Health Institute, American University of Beirut, Beirut, Lebanon; 2 Alice Ramez Chagoury School of Nursing, Lebanese American University, Byblos, Lebanon; 3 Department of Psychology, American University of Beirut, Beirut, Lebanon; 4 Department of Reproductive Health and Research (RHR), World Health Organization (WHO), Geneva, Switzerland; 5 Faculty of Health Sciences, American University of Beirut, Beirut, Lebanon

**Keywords:** Health, Refugees, Sexual and reproductive health, MISP, Migration, Syria

## Abstract

**Background:**

Women and girls are disproportionately affected in times of conflict and forced displacement, with disturbance in access to healthcare services leading to poor sexual and reproductive health outcomes. The minimal initial service package (MISP) was created to mitigate the consequences of conflict and prevent poor sexual and reproductive health (SRH) outcomes, especially among women and girls. The aim of this narrative review was to explore the SRH response for Syrian refugee women and girls in Lebanon, with a focus on MISP implementation.

**Methodology:**

A comprehensive literature search was conducted for peer-reviewed articles in 8 electronic databases and multiple grey literature sites for articles published from March 2011 to May 2019. The target population was Syrian refugee women in Lebanon displaced from Syria as a result of the conflict that erupted in March 2011. The selected articles addressed MISP, SRH needs and services, and barriers to service access. A narrative synthesis was conducted, guided by the six main objectives of the MISP.

**Results:**

A total of 254 documents were retrieved, from which 12 peer-reviewed articles and 12 reports were included in the review. All identified articles were descriptive in nature and no studies evaluating MISP or other interventions or programs were found. The articles described the wide range of SRH services delivered in Lebanon to Syrian refugee women. However, access to and quality of these services remain a challenge. Multiple sources reported a lack of coordination, leading to fragmented service provision and duplication of effort. Studies reported a high level of sexual and gender-based violence, pregnancy complications and poor antenatal care compliance, and limited use of contraceptive methods. Very few studies reported on the prevalence of HIV and other STIs, reporting low levels of infection. Multiple barriers to healthcare access were identified, which included system-level, financial, informational and cultural factors, healthcare workers.

**Conclusion:**

This study highlights the main SRH services provided, their use and access by Syrian refugee women in Lebanon. Despite the multitude of services provided, the humanitarian response remains decentralized with limited coordination and multiple barriers that limit the utilization of these services. A clear gap remains, with limited evaluation of SRH services that are pertinent to achieve the MISP objectives and the ability to transition into comprehensive services. Improving the coordination of services through a lead agency can address many of the identified barriers and allow the transition into comprehensive services.

## Plain English summary

Women and girls are disproportionately affected in times of conflict, with disturbance in access to healthcare services leading to poor sexual and reproductive health outcomes among the vulnerable population. To address the challenges faced by women and mitigate the risks on their health, the minimal initial service package (MISP) was created that outlines the minimum services that should be provided during a humanitarian crisis. The aim of this review was to explore the sexual and reproductive health response in the context of the Syrian refugee crisis in Lebanon.

We identified 12 peer-reviewed articles and 12 reports to be included in this review. The articles described the wide range of services delivered in Lebanon to Syrian refugee women. Despite the multitude of services provided, the humanitarian response remains scattered with limited coordination between agencies and service providers. Studies reported a high level of sexual and gender-based violence, pregnancy complications and poor antenatal care compliance, and limited use of contraceptive methods among Syrian refugee women. Multiple barriers were identified at different levels that restricted healthcare access among this population. Barriers include scattered services, distance and transportation, financial constraints, negative interactions with healthcare workers, misinformation, and cultural barriers.

There is a need to transition into more comprehensive sexual and reproductive health services. Improving the coordination of these services through a leading agency can address many of the identified barriers and improve the sexual and reproductive health of Syrian refugees.

## Background

The Syrian conflict, now in its 9th year, has resulted in hundreds of thousands of deaths and injuries, and more than 5.6 million individuals seeking refuge, mostly in neighboring countries [1]. Lebanon, as of January 2020, hosts around 910,000 registered Syrian refugees, making it the country with the largest number of refugees per capita [2]. Since the start of the crisis, the Lebanese government did not have a clear policy regarding the displaced Syrian population [3]. Lebanon is not a signatory to the 1951 Convention on the Status of Refugees, and the government emphasizes its stance by labeling Syrians as “displaced” [4]. As a matter of fact, the government prevented the establishment of formal refugee camps, leading to the dispersal of refugees throughout Lebanon. The majority of refugees are concentrated in the most impoverished areas with already fragile health infrastructure and scarce services [4]. Many of these refugees reside in informal tented settlements (ITS) and unfinished buildings, which further increases their vulnerability and hinders their access to services [5].

In response to the humanitarian crisis in Lebanon, the Ministry of Public Health (MoPH), the United Nations High Commissioner for Refugees (UNHCR) and other international agencies have been providing health services and humanitarian aid to refugees through several delivery channels [6]. This has resulted in parallel operating systems, including Primary Healthcare Centers (PHCs) operated by the MoPH or Non-Governmental Organizations (NGOs), private clinics and informal healthcare providers, resulting in duplication of efforts and fragmentation of the response [3]. In return, the MoPH called for an integrated approach for health delivery and established a steering committee that would coordinate the planning, financing and delivery of services among the international agencies [3]. Moreover, under the leadership of the MoPH, a network of 220 PHCs across Lebanon have been trained and equipped to provide services to Lebanese and refugee populations [2].

Women and girls are especially vulnerable in times of conflict, with lack of access to services and collapse of social structures leading to poor sexual and reproductive health (SRH) outcomes [7, 8]. The consequences are exacerbated in the case of pregnant women, with a greater risk of premature labor, antenatal complications and infections [8]. In Lebanon, studies have shown that Syrian refugee women are at increased risk of sexual and gender-based violence (SGBV), reproductive tract infections (RTIs), and pregnancy complications [7, 9].

In order to address the disruption in access to basic SRH services, the Inter-Agency Working Group on Reproductive Health in Crisis Settings (IAWG) developed the Minimum Initial Service Package for Reproductive Health in Crisis (MISP) [10, 11]. In 1999, the newly established IAWG released the *Reproductive health in refugee situations: an inter-agency field manual* that formulated a minimum set of interventions, the MISP, to respond to the needs of refugee and displaced populations [10]. MISP defines the core package of services that should be implemented at the onset of an emergency in order to provide the minimum SRH interventions necessary to prevent mortality and morbidity, particularly among women and girls [10]. The MISP objectives in emergency settings are: (1) identifying an agency to lead its implementation, (2) preventing and managing the consequences of sexual violence, (3) reducing Human Immunodeficiency Virus (HIV) transmission, (4) preventing excess maternal and neonatal morbidity and mortality, (5) preventing unintended pregnancies, and (6) planning for comprehensive services and their integration into existing services [10]. The MISP is an emergency response that should be implemented at the onset of a humanitarian crisis, with a plan to scale up to comprehensive SRH services [10]. The recommended services are evidence-based interventions to be implemented in all types of humanitarian crises and to all affected populations [10]. The initial step for the success of the response and implementation of MISP is the identification of a lead SRH agency and coordinator, who in turn provide technical expertise and guidance for the implementation of the subsequent MISP objectives. Following the acute emergency response and the implementation of the MISP objectives, a transition into comprehensive SRH services should be planned and executed [11].

In Lebanon, despite the coordination and efforts of multiple governmental and non-governmental agencies working on the Syrian response, the implementation of MISP and scale up to comprehensive SRH services was limited [7]. The aim of this review is to explore the extent of SRH services implemented in the context of the Syrian refugee crisis in Lebanon, with focused attention on MISP implementation. We will also describe/highlight the availability of different types of SRH services and barriers commonly faced by Syrian refugees in accessing those services.

## Methodology

### Search strategy

A comprehensive literature search was conducted from the start of the conflict in 2011 until May 2019. The search included published peer-reviewed articles, reports and grey literature. Peer-reviewed articles were searched in the following databases: PubMed, Academic Search Ultimate, CINAHL Complete, ERIC, MEDLINE Complete, PsycINFO, Popline, and Global Index Medicus. The review included all types of study designs: case–control, cohort, case-studies, cross-sectional and qualitative studies. The following MESH terms and keywords were used for this search: “Syrian”, and “Refugees” and “Lebanon,” combined with the following terms “Minimum Initial Services Package”, “Inter-agency Field Manual”, “Women”, “Girls”, “Maternal”, “Health”, “HIV”, “Emergency System”, “Response Plan”, “Displaced population”, “Sexually Transmitted Infections (STIs)”, “Antenatal Care (ANC)”, “Violence”, “Child Marriage”, “Access Services”, “Contraception”, “Obstetric”, “Reproductive”, “Sexual”. A search of the grey literature was conducted for reports and commentaries from credible humanitarian organizations and databases, including RefWorld, ReliefWeb, and UNHCR. These sources were selected based on the inclusion and exclusion criteria (Table 1), and the experience of authors/organizations in SRH needs of Syrian refugees. The broad nature of the search strategy was utilized to allow us to access all relevant literature that may include information on MISP implementation or SRH services for Syrian refugees in Lebanon.

**Table 1:** Inclusion and exclusion criteria

Category	Included	Excluded
Population of interest	Syrian refugee women receiving MISP or other SRH services and interventions in Lebanon	Studies focusing on other refugee populations in Lebanon or those that did not address SRH services and needs among Syrian women
Health outcomes	Primary outcomes (prevention of SGBV, maternal mortality and morbidity, HIV and STI prevention). Secondary outcomes (response to needs of SGBV survivors, prevention of unintended pregnancies)	Studies which did not quantify the MISP and/or SRH-related health indicators
Intervention	Interventions and programs seeking to improve SRH outcomes in Lebanon	
Humanitarian crisis	Studies on the Syrian refugee crisis in Lebanon	Syrian refugees residing in neighboring countries, those displaced prior to the civil war (2011), Palestinian refugees from Syria, and other refugee populations in Lebanon
Study design	All study designs (quantitative, qualitative, mixed methods)	
Publication type	Peer reviewed studies and reports from international agencies	Manuscripts that had not undergone peer-review, such as dissertations and conference abstracts
Publication date	February 2011 to May 2019	
Language	English	

### Eligibility criteria

Included in this review are studies conducted in Lebanon addressing the SRH response, needs, and barriers of Syrian refugee women. Inclusion and exclusion criteria were based on the Inter-Agency Field Manual on reproductive health in humanitarian settings [11] as outlined in Table 1.

### Data collection and syntheses

**Fig. 1:**
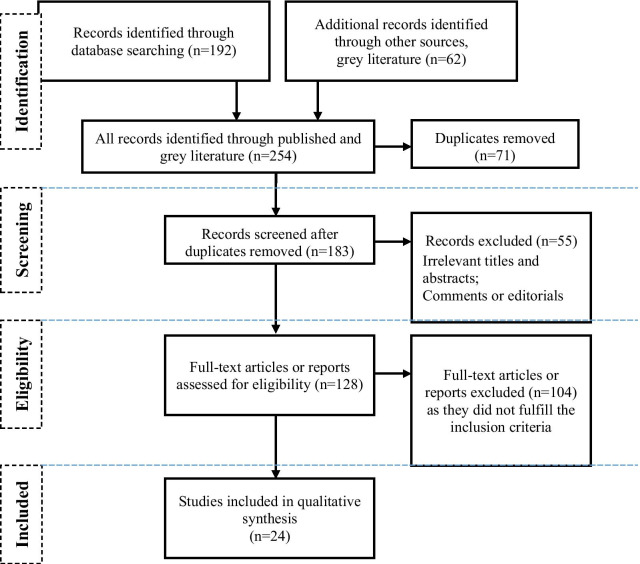
Adapted the preferred reporting items for systematic reviews and meta-analyses (PRISMA) flow chart

Following the screening of the title, abstract and full text of the identified articles, data for this review were extracted from the included studies. The extraction focused on the following data: participant characteristics, research setting, health outcomes, intervention description, key findings and recommendations. Given that the majority of studies were qualitative, we synthesized and reported the findings narratively (Fig. 1).

## Results

A total of 254 documents were retrieved and screened, of which 12 peer-reviewed articles and 12 reports from the grey literature were included in the results (Table 2). None of the articles evaluated interventions or programs. All articles extracted from the literature were descriptive in nature: qualitative and quantitative studies addressing the current situation, prevalence, services available and barriers. Though a few studies were specific, the majority addressed multiple SRH domains. The results are summarized in a narrative manner and were analyzed using a focused synthesis around the six MISP objectives highlighting: current state, response and services presented under each objective. This is followed by a summary of the reported barriers to accessing these services.

**Table 2:** Characteristics of publications included in the review

Authors	Article	Year	Publication type	Participant characteristics	Summary of relevant findings
*Peer-reviewed articles*					
Kabakian-Khasholian et al. [5]	Perspectives of displaced Syrian women and service providers on fertility behaviour and available services in West Bekaa, Lebanon	2017	Qualitative study	84 married Syrian refugee women	Changing fertility patterns following displacement Barriers to SRH services and contraceptive use: high cost, discrimination, lack of women providers
Samari et al. [7]	Syrian refugee women's health in Lebanon, Turkey, and Jordan and recommendations for improved practice	2017	Review	N/A	Poor access to SRH services and decreased use of contraception post displacement Barriers to access include high cost, distance and mistreatment
Benage et al. [8]	An assessment of antenatal care among Syrian refugees in Lebanon	2015	Cross-sectional study	420 pregnant Syrian refugee women	Only 15.7% of women received the required 4 antenatal care (ANC) visits. The quality of ANC services was subpar with 44.6% of those attending 4 or more visits receiving all 3 necessary interventions
Masterson et al. [9]	Assessment of reproductive health and violence against women among displaced Syrians in Lebanon	2014	Cross-sectional study	452 Syrian refugee women	High prevalence of pregnancy complications (39.5%) and adverse birth outcomes High level of SGBV with few seeking care
Mourtada et al. [17]	A qualitative study exploring child marriage practices among Syrian conflict-affected populations in Lebanon	2017	Qualitative study	24 Adolescent Syrian girls and 26 parents	Higher incidence of early and forced marriage in Lebanon following displacement
Usta et al. [18]	Violence against displaced Syrian women in Lebanon	2019	Qualitative study	29 Syrian refugee women	SGBV was common in multiple settings including interpersonal violence
DeJong et al. [20]	Reproductive, maternal, neonatal and child health in conflict: a case study on Syria using Countdown indicators	2017	Review	N/A	Low antenatal care utilization, high C-section rate
Tohme et al. [23]	HIV prevalence and demographic determinants of unprotected anal sex and HIV testing among male refugees who have sex with men in Beirut, Lebanon	2016	Cross-sectional study	213 men, of which 48 were refugees	Low levels of HIV prevalence and testing
Tappis et al. [24]	Maternal health care utilization among Syrian refugees in Lebanon and Jordan	2017	Cross-sectional study	1376 Syrian refugee households	Majority of women reported seeking ANC with an average of 4.8 visits. High C-section rate. Barriers to access include high cost, long waiting lines and restricted movement
Huster et al. [24]	Cesarean sections among Syrian refugees in Lebanon from December 2012/January 2013 to June 2013: probable causes and recommendations	2014	Mixed methods	6,366 delivery records and 9 refugee women interviewed	Deliveries accounted for 50% of hospitalizations and C-section rate was 35%
Cherri et al. [28]	Early marriage and barriers to contraception among Syrian refugee women in Lebanon: a qualitative study	2017	Qualitative study	108 Syrian refugee women	Barriers to contraceptive use: lack of preferred method, cost, perceived side effects and misinformation
Talhouk [29]	Syrian Refugee and Digital Health in Lebanon: Opportunities for Improving Antenatal Health	2017	Qualitative study	59 Syrian refugee women	Barriers to antenatal care access: distance, cost, discrimination
*Grey Literature*					
MOPH [2]	Health Response Strategy: Maintaining Health Security, Preserving Population Health & Saving Children and Women Lives	2016	Report	N/A	The MoPH was leading and strengthening the healthcare response to the Syrian refugee crisis through its network of PHCs and specialized reproductive health services
UNHCR [12]	Sexual and Gender-based Violence Prevention and Response **i**n Refugee Situations in the Middle East and North Africa	2015	Report	N/A	Increased exposure to SGBV among Syrian refugees
UNFPA [13]	Independent Country Evaluation Programme: Lebanon	2014	Report	N/A	Throughout 2012, UNFPA conducted 4 MISP trainings to healthcare workers
UNFPA [14]	Key results of Lebanon in 2018	2018	Report	N/A	120 healthcare workers received MISP training in 2018
Anani [15]	Dimensions of gender-based violence against Syrian refugees in Lebanon	2013	Report	Unspecified	Higher level of SGBV following displacement and scattered services
Essaid et al. [16]	Gender based violence against women and girls displaced by the Syrian conflict in south Lebanon and north Jordan: Scope of violence and health correlates	2015	Mixed methods study	182 Syrian refugee women in Lebanon	High level of experienced violence following displacement with violence associated with poor utilization of services and SRH outcomes
Amnesty International [19]	‘I want a safe place’ Refugee women from Syria uprooted and unprotected in Lebanon	2016	Report	65 Syrian refugee women	SGBV was common with multiple barriers to accessing care including legal status and movement restrictions
UNHCR [21]	Consultative meeting on Clinical Management of Rape (CMR) Services	2016	Presentation	N/A	Limited service provision at some CMR centers due to lack of trained staff
K2P [22]	Addressing Limitations to Equitable Access to Healthcare Services for People Living with HIV in Lebanon	2015	Review	N/A	Despite the presence of Voluntary Testing and Counselling (VCT) centers in Lebanon, the majority are not functioning
K2P [25]	Reducing preventable Preterm Deliveries among Syrian Refugees in Lebanon	2017	Review	N/A	High rate of C-section, delivery complications and neonatal mortality. Poor utilization of postnatal care
UNHCR [26]	Health access and utilization survey among Syrian refugees in Lebanon	2017	Cross-sectional study	479 households	The majority of women received antenatal care during pregnancy. 31% of women delivered by C-section. The most commonly used contraceptive method was OCP
Chahine et.al [30]	Situation analysis of youth in Lebanon affected by the Syrian crisis	2014	Mixed methods	985 Syrian refugee youth	Poor knowledge of contraception among Syrian refugee youth, including those who are married

## MISP implementation and SRH services in Lebanon

### Coordination and Lead on implementation of the response

Starting in 2015, the MoPH and UNHCR established a committee to coordinate and guide activities in coordination with the Health Working Group, under which there is a sub-working group for Reproductive Health and SGBV that is led by UNFPA [2, 12]. The Lebanon Crisis Response Plan (LCRP) 2015–2016 was developed to strengthen the coordination and leadership role of the government in the humanitarian response, followed by a 2017–2020 plan [2]. The MoPH, with support from UNHCR and UNFPA, strived to strengthen the PHC response, with some of these centers providing specialized reproductive health services [2]. However, the challenge remains due to the lack of a national reproductive health strategy, despite the ongoing efforts of WHO and UNFPA [13].

MISP trainings have been undertaken in Lebanon under UNFPA, as part of its capacity development interventions to enhance the ability of Healthcare Workers (HCWs) to respond to humanitarian emergencies [13]**.** Throughout 2012, UNFPA conducted four trainings on MISP for HCWs in collaboration with the Lebanese government and the Lebanese Society for Obstetrics and Gynecology [13]. However, in spite of the support of MoPH, the lack of resources limited the scale-up and sustainability of these trainings [13]. Nonetheless, as per the 2018 UNFPA report, 120 HCWs received MISP training in 2018 [14].

### Prevent sexual violence and respond to the need of survivors

In regards to SGBV, different measures were put in place by several national and international NGOs. These included prevention and protection programs through legal services, hotline services, protection and shelter, and medical and psychological health services to all survivors of SGBV covered for free [7, 12, 15]. For example, Abaad NGO in collaboration with UNHCR, UNICEF and the Danish Refugee Council opened three safe shelters for Syrian refugee women at risk of SGBV, providing housing, case management, counseling, psychosocial and legal support, and medical care [15]. According to UNHCR, it has been estimated that two thousand Syrian refugee women benefit each month from services provided at safe spaces [12].

A number of articles highlighted the high prevalence of SGBV among Syrian refugee women in Lebanon, however, quantitative studies and accurate estimates remain lacking [7, 12, 15]. A study conducted by Essaid in 2015, with a sample of 182 Syrian refugee women from South Lebanon, found that 26% of Syrian refugee women experienced emotional violence, 9.2% physical violence, and 8.7% sexual violence, with many women reporting exposure to more than one type of violence [16]. Syrian women reported increased exposure to multiple forms of SGBV, including sexual assault, domestic violence, child marriage and sexual exploitation [12, 15]. However, only 35% of Syrian refugee women who experienced violence sought medical care, and 9.2% psychosocial assistance [8, 9].

Increase in sexual assault was associated with living in insecure areas with little privacy and breakdown of existing social networks [15, 17]. Syrian refugee women believed sexual violence was fairly common, and they feared abuse in multiple settings and by different perpetrators, including guards at checkpoints, landlords, employers, shop keepers and taxi drivers [16, 18, 19]. As a result, some women avoided walking unaccompanied in the streets [18]. Moreover, due to the illegal status of many refugees in Lebanon, men fear checkpoints and so prefer to send their wives to receive assistance, as they are less likely to be stopped and detained. This is believed to have increased the risk of sexual harassment and exploitation at sites of aid distribution or on the way towards such centers [15]. Sexual exploitation, or “survival sex”, has also been reported among Syrian refugee women and was associated with the need to cover the cost of living, i.e. in exchange for food, rent and other goods and services [15]. A few participants in the qualitative arm of the study reported that husbands coerce their wives into sexual relations with others for money [16].

Syrian refugee women reported an increase in domestic violence since their displacement. This was perceived to be due to increased frustration among Syrian men, leading them to release their anger on their wives [15, 16, 18]. Many reasons were reported to contribute to this, including their refugee status, mistreatment by employers, and loss of their role as providers for the family [15, 16, 18]. One study reported how women are still expected to fulfill their traditional roles of household management, cooking and childcare [15]. The inability of women to fulfill their roles due to financial constraints and poor living conditions results in conflict between the spouses [16]. In the same study, 15% of women reported being afraid of their husbands most of the time [16].

Forced and early marriage appears to be increasing among refugees in Lebanon in comparison to pre-conflict rates in Syria [20]. Studies estimate a prevalence of 18 to 23%, compared to the previous 17.3% in pre-conflict Syria [17, 20]. Qualitative studies on child marriage show that Syrian families are accepting an earlier age for marriage due to loss of educational opportunities, financial constraints, and protection of girls from sexual harassment [15, 16, 20].

SGBV has also been significantly associated with poor SRH outcomes among Syrian refugee women, including lack of ANC uptake, and preterm delivery [16]. A minority of Syrian refugee women, in a qualitative study, also reported increased risks of STIs and/or spontaneous abortions as a result of the violence [16]. Moreover, a study conducted by Masterson et al. showed a significant correlation between exposure to violence and gynecological conditions, such as menstrual irregularity, severe pelvic pain and RTIs [9].

Many challenges remain in the response to SGBV among Syrian refugees in Lebanon. UNHCR data shows that although CMR is established across Lebanon and referral systems are in place, 13% of the assessed centers could not perform their tasks because of high staff turnover, lack of human resources or poor training [21]. Other problems exist such as the limited awareness by the police of the relevant referral pathways, break of confidentiality and lack of post-exposure prophylaxis kits to name a few [21]. Furthermore, lack of trust in Lebanese authorities and the difficulty of obtaining legal status hinder reporting and seeking support following sexual assault [19].

### Prevent the transmission of and reduce morbidity and mortality due to HIV and other STIs

Limited data is available around the epidemiology and burden of HIV and other STIs among Syrian refugees in Lebanon. In 2015, it was estimated that a total of 1893 people are living with HIV in Lebanon. This figure includes both Lebanese citizens and refugees [22]. Only one study was found on refugees living with HIV in Lebanon. This study, by Tohme et al., was conducted in 2016 among 150 Syrian, Iraqi and Palestinian male refugees who have sex with men [23]. The study results showed that 2.7% of the participants were infected with HIV, and 60% of the participants reported a lifetime STI, with the most common ones being: human papillomavirus (28.7%), gonorrhea (24%), chlamydia (23.3%) or genital lice (18%) [23].

It is important to note that Lebanon has an established National AIDS Control Program (NAP) that provides free Antiretroviral Treatment to all eligible patients, including registered refugees [22]. NAP in collaboration with NGOs is involved in a number of interventions, namely awareness campaigns and outreach programs, establishing Voluntary Counselling and Testing centers and training staff [22]. However, most of the available 110 voluntary counseling centers spread across Lebanon are not functioning, and services like CD4 count, viral load and Chest X-ray are not available for free [22].

### Prevent excess maternal and newborn morbidity and mortality

It has been estimated that between 20 and 40% of Syrian refugee households in Lebanon have at least one pregnant woman [24]. According to one study among 452 Syrian refugee women of reproductive age, 9.5% were currently pregnant [9]. Moreover, 57% of UNHCR referrals and hospitalizations are pregnancy-related and approximately 40% of UNHCR’s expenditure is used for maternal care [2, 25]. Services covered by UNHCR for registered refugees include ANC visits at a reduced price, coverage of 85% of laboratory fees and two free ultrasounds [5, 8], and coverage of $250 for normal vaginal delivery, and $500 for C-Sect. [25].

The current Lebanese MoPH recommendation is that pregnant women should attend at least 4 ANC visits. However, in a study conducted among 420 pregnant Syrian refugee women, 17.1% did not access ANC, 66.1% accessed 1 to 3 ANC visits, and only 15.7% received all 4 required consultations, compared to 64% in Syria prior to the conflict [8]. Another study reported a similar rate, with 20% of women not receiving ANC in their most recent pregnancy [16]. However, Tappis et al. reported 88.7% of women receiving ANC care with an average of 4.8 ANC visits [24]. However, the study showed that 45% of women did not receive ANC during their first trimester [24]. And a study by UNHCR in 2018, demonstrated that although 70.5% of Syrian refugee women sought ANC, 30.7% reported difficulties in accessing the services [26]. Benage et al. assessed the quality of ANC services by assessing whether the Syrian refugee women received the three required components of ANC, namely blood sample analysis, urine analysis, and blood pressure measurement. Among women who received all 4 ANC consultations, only 44.6% received these three basic interventions [8]. Moreover, 90.5% of women did not receive the tetanus prophylaxis vaccine, and 42.1% were not educated about pregnancy danger signs [8].

There is also a high level of pregnancy and delivery-related complications among Syrian refugee women, despite the fact that almost all deliveries are attended by skilled birth attendants [9, 20, 26, 27]. Masterson and colleagues reported that 39.5% of pregnant women in their sample reported experiencing pregnancy complications [9]. Clinicians in a qualitative study by Huster et al. in 2013, reported that a significant amount of pregnancy complications, associated with poor ANC compliance, are diagnosed only at delivery often requiring emergency C-sections [20, 27]. It should also be noted that the C-section rates among Syrian refugee women are much higher than the recommended WHO limit of 15%. Some studies report rates ranging between 30.6 and 35% [20, 24–26]. Furthermore, up to 61% of hospital mortality among Syrian refugees is among children under one year of age, with 25% of these deaths the result of preterm delivery [2, 25].

### Prevent unintended pregnancies

Free family planning services and contraception are provided to Syrian refugee women through multiple programs [5, 8]. However, a number of studies demonstrate poor contraceptive use (between 42.3 and 65.5%) among Syrian refugee women [8, 9]. Although, it was reported that 74% of Syrian refugee women wanted to prevent a future pregnancy and 52% of women did not desire their current pregnancy [8]. These low rates of contraceptive use among Syrian refugee women in Lebanon are in contrast with the nationally reported rates in Syria before the crisis, where 58 to 73% of Syrian refugee women were using some contraceptive method [5, 9]. In Lebanon, the most commonly used contraceptive methods were: the Intrauterine Device (IUD):19.6%, followed by Oral Contraceptive Pills (OCPs): 8.6%, and periodic abstinence: 3.5%. In a more recent study conducted by UNHCR in 2018, among 194 interviewed women, 38% reported OCPs to be the most frequent method of contraception, followed by IUDs (31%%), traditional methods (25%), and lastly condoms (13%) [26]. These most commonly used contraceptive methods are similar to those reported in pre-conflict Syria [9, 28].

Syrian refugee women narrate their desire for large families, with 4 to 6 children being the desired family size, similar to the average of 4.9 desired children in pre-conflict Syria [5]. It has been cited that refugee women tend to desire more children in order to compensate for the death rates in the war, to maintain a gender balance within the family or to have a boy to support the family [5]. In addition, very low rates of contraceptive use are observed among newly married couples prior to their first pregnancy, and/or prior to achieving their desired family size and/or having the first male child [5, 28]. However, lately, it is being observed that younger women (aged 18–25 years) are changing their attitude as a result of the conflict, with some wanting to limit the number of children to 1 or 2. This is associated with the prevailing financial and legal difficulties they are facing [5]. Many women reported resorting secretly to family planning, while their husbands were in favor of increasing the family size [5].

### Plan for comprehensive reproductive health services

Access to reproductive health services is available to Syrian refugee women through the national PHC network, at a subsidized consultation cost [5]. Yet, even after 9 years since the start of the refugee crisis, access and utilization of SRH services remain limited. According to Masterson et al., only a third of the women in the survey conducted in 2014 perceived that SRH services were accessible, and only a quarter of the surveyed women reported vising a gynecologist in the past 6 months [9]. In fact, the majority of women in the survey reported that they only visit a gynecologist when they are pregnant [9].

## Barriers to accessing SRH services

Multiple barriers have been associated with the poor utilization of SRH services, these can be summarized into system-related and process, HCWs, financial, informational and cultural barriers, as detailed below.

### System-related and process barriers

The review of the literature identified a number of system-related barriers to SRH service access. First of all, SRH services are decentralized and scattered across Lebanon [15]. This is particularly true when it comes to SGBV services. Women often have to access the different services needed through different providers at different access points, thus limiting their ability to access all the services they need comprehensively [15]. There is also minimal coordination among NGOs and the different service providers, often resulting in a lack of clear referral systems and duplication of efforts [15]. Many services are also only provided to registered Syrian refugee women, which excludes the most vulnerable population of women who are unregistered in Lebanon [16]. Mistrust in the quality of SRH care provided is another important challenge, as not all SRH services provided at the health facilities have equivalent quality standards [8].

Distance to services and PHCs is another major barrier. Around 16 to 25% of Syrian refugee women reported transportation as an important impeding variable to access services [9, 16]. Talhouk et al. described how ANC follow-up frequency depends on the distance to PHC. Women living further away from the PHCs limited their ANC visits to one during the early stages of pregnancy followed by another prior to the estimated delivery date [29]. Syrian refugee women living in remote areas and settlements are also less likely to receive awareness sessions provided by NGOs, equally due to their inability to leave the settlements and unaffordable transportation costs [17].

Moreover, few studies reported the limited availability of the free necessary SRH supplies, which quickly run out of stock due to the high demand [5, 16]. This was specifically true for prenatal vitamins and contraceptives [5, 16]. When specifically analyzing the barriers to contraception use, lack of preferred method (compared to availability in pre-conflict Syria) was a prominent finding [9, 28]. For instance, many Syrian refugee women were unable to obtain injectable contraception in Lebanon, a method preferred by many in pre-conflict Syria, a concern also frequently echoed by HCWs [5, 28].

Another frequently cited system-related barrier was the negative experiences with the health care providers. According to a qualitative study conducted by Talhouk et al. in 2015, one of the reasons why Syrian women did not receive the desired number of ANC visits was their negative interactions with healthcare providers [29] as well as the long wait times [24]. This has resulted in the commonly reported lack of trust in the Lebanese healthcare providers [16].

Furthermore, access to healthcare services in certain areas is impeded by security concerns [9, 16]. Legal status and documentation obstruct women’s access to healthcare services as they fear passing through checkpoints, detention and imprisonment [19].

### Healthcare workers

Lack of trained staff and HCWs was reported as one of the main reasons for not accessing SRH services by almost 20% of women [9, 16]. This is especially true when it comes to the availability of women HCWs, which is a common preference by Syrian refugee women [5, 9].

The literature cites an extensive number of negative interactions with HCWs and staff experienced by Syrian refugee women when accessing healthcare [5, 9, 15–17, 29]. These include little time spent during patient consultations coupled with little medical information or explanations provided, prejudice against Syrian refugee women, as well as discrimination and mistreatment [9, 16, 29]. As a result, some women avoid visiting healthcare facilities [15, 29], mainly due to fear of mistreatment (as indicated by around 8% of Syrian refugee women) [9]. This is especially true for women who experienced SGBV, limiting and/or preventing access to the needed healthcare and psychosocial support [15, 16].

### Financial barriers

Cost is a significant barrier to accessing SRH services. One survey showed that only 24–36% of Syrian refugee women indicated that SRH services were affordable and accessible [16]. In the study by Masterson et al. in 2014, cost was the main barrier to access SRH services, as reported by 49.7% of women [9]. In addition, financial concerns continue to be an important impediment to contraceptive use [5, 9, 16, 29].

Despite the re-enforced subsidies to access PHC services, cost of care remains the main barrier to accessing ANC, as reported by 76–78% of the Syrian women [16, 24]. Transportation fees and medications are not covered, which add up to an additional cost of 20–30 USD per visit [5, 17].

### Information barriers, misconceptions and difficulties in navigating the system

Limited awareness is another important factor, as a significant proportion of women do not know what services are available, which are subsidized or free, and how and where they could seek access to these services [15, 16]. It was observed that up to 37.8% of Syrian refugee women in Lebanon did not know that free or subsidized SRH services were available. For example, one of the main barriers to contraceptive use among this population was the lack of knowledge of where they could access different contraceptive methods for free [28]. The same pattern was observed when seeking ANC during pregnancy, as 16.1% of the women were not aware that these services were subsidized in PHCs [25]. Postnatal care (PNC) is even more underutilized by Syrian refugee women due to the lack of knowledge about its availability, with only 26% of registered refugees accessing postnatal care [25].

The lack of adequate knowledge was often associated with false misconceptions. For instance, Syrian women avoid OCPs due to their perceived side effects [5, 16, 28]. It is widely believed that taking OCPs prior to the first pregnancy can cause infertility [5, 28]. Some Syrian women also do not access ANC because they are not aware of the benefits and believe that they do not need them, up to 32% in one study [16, 25]. This knowledge gap is even more prominent among the youth. A survey among adolescent Syrian refugees led by UNHCR in 2014 showed that 66% of adolescent girls and 63% of adolescent boys had poor knowledge of different family planning methods, even among married youths, 18% of whom lacked information about family planning [30].

### Cultural barriers

Cultural factors play a very important role in access to SRH services, especially family planning. The decision regarding the use of contraception and the number of children to have is most often made by the husband [5, 29]. In many cases, women cannot negotiate the use of contraception to delay childbirth, especially in the case of a recent marriage with everyone waiting for the first child to arrive [28]. Women, as well, fear polygamy or replacement in case they refused to bear more children [5, 28]. This is further challenged with the fact that most awareness campaigns only target women and girls [17].

In addition, restricted movement of women for safety concerns hinders access to SRH services [16]. In a study conducted by Tappis and colleagues, 10.3% of women could not access ANC due to lack of male permission [24]. Restricted movement is especially prominent in the cases of access to SGBV services, where women may not be able to access services either because they are often accompanied by their husbands or another family member [15, 16]. Survivors of sexual violence are also unable to access services or report the incidents due to the fear that they will “bring dishonor to the family” [15, 16].

## Discussion

The results from this narrative review highlight the breadth of literature in Lebanon around practices, access and use of, and barriers to SRH services experienced by Syrian refugee women in Lebanon. It is clear that there is a high interest among the international and local humanitarian community in addressing SRH in emergencies, as evident by the wide range of SRH services delivered in Lebanon. However, there is a lack of systematic MISP implementation in Lebanon in response to the Syrian refugee crisis, and access and quality of available services remain a challenge.

Moreover, the review highlights the gaps in these services and inadequate coverage leading to an unmet need and reported poor overall reproductive health of Syrian women in Lebanon. A high level of SGBV is reported, mainly sexual harassment in a variety of settings by multiple perpetrators, and early marriage. Moreover, a high pregnancy rate and the unmet need for contraception have been highlighted by the presented studies. In addition, Syrian refugee women experience a high level of pregnancy complications and C-section rates placing a great burden on the system. There are very few studies investigating the prevalence of HIV and other STIs. However, Lebanon has an overall low level of HIV and a coordinated response by the NAP. These poor reproductive health outcomes are the result of displacement and poor access to healthcare services.

The reviewed articles described how the limited access to SRH services in Lebanon is due to the overall system and architecture of service provision, and a number of individual factors. The humanitarian SRH services architecture is decentralized, provided by multiple actors with limited coordination, leading to non-complementary services and narrow reach to the affected population. Syrian refugees’ mistrust of HCWs and experience of discrimination and ill-treatment are a common barrier to their utilization of services in Lebanon. It has been found that the application of SRH knowledge is largely affected by the trust of the patients in HCWs and the programs [31]. Several trainings were given to service providers; however, the trainings focus on building the knowledge of the trainees, with no follow up on the adoption of the skills, and integration in the processes of the health system. Systematic reviews on the effectiveness of SRH interventions in humanitarian settings demonstrated strong support for community HCWs, peer-led education and counseling, and moderate evidence for transport-based referral systems [31]. These interventions, along with a leading agency to improve coordination, provide a solution to many of the barriers present in Lebanon and may lead to better utilization of services.

Despite multiple interventions and programs being implemented, no studies have been conducted on the effectiveness of different programs or interventions. A clear gap remains around the evaluation of services that are pertinent to the MISP objectives and the ability to transition from MISP to comprehensive SRH services after nine years from the onset of the crisis. No studies were identified evaluating the context of SRH service delivery among this target population and particularly around evaluating MISP implementation during the acute stages of this crisis. A similar gap in studies evaluating MISP and SRH interventions was found by Col et al. in Turkey, with only one interventional study identified in their recent review [32].

The MISP package for reproductive health in crisis was developed over two decades ago to respond to the SRH needs of women and girls in times of emergencies. The first global evaluation of the MISP in 2004 demonstrated a low level of awareness among HCWs coupled with a clear lack of systematic implementation [33]. In 2018, the IAWG introduced a number of revisions to the MISP, most notably adding unintended pregnancy prevention as a separate objective to highlight its priority and a stronger emphasis on a transition into comprehensive SRH services [10]. To date, there remains a limited number of studies evaluating the implementation and delivery of MISP in the context of different humanitarian crises, and the evidence around its effectiveness remains scarce [33]. As per the review, there is limited MISP implementation in Lebanon, with only a few trainings conducted at the beginning of the crisis and no leading agency or coordinator appointed to implement the MISP package. Such a lack of coordination has been observed in a number of countries where MISP implementation was evaluated [34]. Lack of coordination was also found to lead to poor communication, resulting in shortage or duplication of services [34]. Among Syria’s neighboring countries affected by the refugee crisis, MISP implementation was conducted only in Jordan, facilitated by dedicated leadership and available funding, leading to the availability of a number of services, but only among camp residents [35]. In an assessment of MISP coordination in Eastern Europe and Central Asia, it was also observed that an improvement in coordination at the national level led to better preparedness during the response to the crisis, with noticeable improvements in multiple indicators across the MISP objectives [36].

Though MISP has not been implemented as a package in Lebanon with a clear leading agency and coordinator, multiple SRH services are provided by the formal healthcare system. However, as indicated, multiple barriers impact access to these services, a handful of which could be alleviated by better coordination. Poor referral systems and lack of information about services, absence of preferred contraception method and lack of supplies and trained staff are to name a few. These barriers can be reduced with better coordination and distribution, in order to prevent duplication of services and the absence of others. Moreover, contextualization of SRH interventions is essential, as poor service utilization was associated with a range of cultural barriers. In Jordan, despite coordination and availability of services, poor utilization was associated with limited knowledge of the availability of the different SRH services coupled with negative interactions with HCWs [35].

Planning for and transitioning to comprehensive SRH service delivery is an essential objective of the MISP that was further emphasized in the 2018 revisions [10]. This transition requires the collection of information and planning for integrated, comprehensive and appropriate SRH services. In Lebanon, although multiple services addressing the MISP objective have been implemented as evident by this review, MISP was not implemented comprehensively from the onset of the crisis. Nine years later, in the context of a protracted crisis, there has been no scale-up and limited economic and social support to address the reported barriers. Proper implementation and effective planning for comprehensive services depend on the first objective of the MISP: coordination. The appointment of a lead agency that is aware of the socioeconomic, political and cultural context, and has the credibility and ability to coordinate between the major agencies, service providers and the government is essential if the MISP is to achieve its other objectives [37, 38].

This review was limited to English publications, which may have excluded mostly governmental reports. Due to the nature of grey literature searches, relevant publications may have been missed from international organizations whose websites were not surveyed and were not available on the searched databases. More importantly, most studies were descriptive and qualitative in nature, addressing specific SRH outcomes and services limiting the ability to generalize or compare across studies.

## Conclusion

This review summarizes the humanitarian response to the SRH needs of Syrian refugee women in Lebanon. It highlights the main services provided, their use and access by women and multiple barriers they face that limit the utilization of these services. MISP implementation was not conducted in Lebanon at the start of the crisis, and lack of coordination was highlighted as the main challenge in the response. More research is necessary to evaluate the MISP and its implementation in a different humanitarian setting, in order to establish best practices, cost-effectiveness and identify barriers to its implementation. Our results highlight a general lack of evaluation of interventions and programs on SRH in Lebanon. In order to respond to the needs of the displaced population, an evaluation of the existing programs and interventions is essential to develop evidence-based solutions that would circumvent the barriers commonly experienced by women. Moreover, in the setting of protracted crisis, it is important to transition into the sixth objective of the MISP package, comprehensive and integrated services. In order to do so, strong leadership and coordinating body should be selected.

## Data Availability

Not applicable.

## Abbreviations

MISP: Minimal initial service package
SRH: Sexual and Reproductive Health
MoPH: Ministry of Public Health
UNHCR: United Nations High Commissioner for Refugees
PHC: Primary Healthcare Center
NGO: Non-Governmental Organization
SGBV: Sexual and gender based violence
RTI: Reproductive tract infection
IAWG: InterAgency Working Group
HIV: Human Immunodeficiency Virus (HIV)
STI: Sexually transmitted infection
ANC: Antenatal care
CMR: Clinical Management of Rape
UNFPA: United Nations Population Fund
WHO: World Health Organization
HCW: Healthcare worker
NAP: National AIDS Control Program
IUD: Intrauterine device
OCP: Oral contraceptive pills
